# Serial plasma DNA levels as predictors of outcome in patients with acute traumatic cervical spinal cord injury

**DOI:** 10.1186/s12967-019-2084-z

**Published:** 2019-10-01

**Authors:** Hung-Chen Wang, Yu-Tsai Lin, Shih-Yuan Hsu, Nai-Wen Tsai, Yun-Ru Lai, Ben Yu-Jih Su, Chia-Te Kung, Cheng-Hsien Lu

**Affiliations:** 1 Department of Neurosurgery, Kaohsiung Chang Gung Memorial Hospital and Chang Gung University College of Medicine, Kaohsiung, Taiwan; 2 Department of Otolaryngology, Kaohsiung Chang Gung Memorial Hospital and Chang Gung University College of Medicine, Kaohsiung, Taiwan; 3 Department of Neurology, Kaohsiung Chang Gung Memorial Hospital and Chang Gung University College of Medicine, 123, Ta Pei Road, Niao Sung Dist., Kaohsiung, Taiwan; 4 Department of Medicine, Kaohsiung Chang Gung Memorial Hospital and Chang Gung University College of Medicine, Kaohsiung, Taiwan; 5 Department of Emergency Medicine, Kaohsiung Chang Gung Memorial Hospital and Chang Gung University College of Medicine, Kaohsiung, Taiwan; 6 Department of Biological Science, National Sun Yat-Sen University, Kaohsiung, Taiwan; 7 Department of Neurology, Xiamen Chang Gung Memorial Hospital, Xiamen, Fujian China

**Keywords:** Traumatic spinal cord injury, Plasma DNA, Outcome, Japanese Orthopaedic Association score

## Abstract

**Background:**

Acute traumatic cervical spinal cord injury (SCI) is a leading cause of disability in adolescents and young adults worldwide. Evidence from previous studies suggests that circulating cell-free DNA is associated with severity following acute injury. The present study determined whether plasma DNA levels in acute cervical SCI are predictive of outcome.

**Methods:**

In present study, serial plasma nuclear DNA (nDNA) and mitochondrial DNA (mtDNA) levels were obtained from 44 patients with acute traumatic cervical SCI at five time points from day 1 to day 180 post-injury. Control blood samples were obtained from 66 volunteers.

**Results:**

Data showed a significant increase in plasma nDNA and mtDNA concentrations at admission in SCI patients compared to the control group. Plasma nDNA levels at admission, but not plasma mtDNA levels, were significantly associated with the Japanese Orthopaedic Association (JOA) score and Injury Severity Score in patients with acute traumatic cervical SCI. In patients with non-excellent outcomes, plasma nDNA increased significantly at days 1, 14 and 30 post-injury. Furthermore, its level at day 14 was independently associated with outcome. Higher plasma nDNA levels at the chosen cutoff point (> 45.6 ng/ml) predicted poorer outcome with a sensitivity of 78.9% and a specificity of 78.4%.

**Conclusions:**

These results indicate JOA score performance and plasma nDNA levels reflect the severity of spinal cord injury. Therefore, the plasma nDNA assays can be considered as potential neuropathological markers in patients with acute traumatic cervical SCI.

## Background

Acute traumatic spinal cord injury (SCI) is a major cause of disability among teenagers and young adults in many countries [1]. Primary and secondary injuries are two major pathophysiological causes of neurological deficits [2]. Primary injury refers to an insult that occurs at the time of impact that is not amenable to therapy. Secondary injury results from neuroinflammation or ischemia that lead to cord edema and cellular destruction [3–5]. The extent of spinal cord damage is determined by the severity of the primary mechanical injury and the intensity of the secondary biomolecular injury. Therefore, there is a therapeutic window of opportunity for intervention with respect to secondary injury.

Baseline levels of circulating cell-free deoxyribonucleic acid (DNA) are present in the normal healthy population, albeit at very low levels [6, 7]. Significant increases of cell-free circulating DNA in the plasma of trauma patients have been reported and correlated with severity of injury and development of post-traumatic complications [8–13]. Otherwise, elevated levels of plasma DNA have been shown to be associated with cell death in various diseases, including various infections [14–16], cancer [7, 17], and vascular events [18–24]. Cells tend to experience apoptosis or cell death due to impairment of the mitochondrial function following SCI [25–28]. This process releases cell-free DNA, including mitochondrial DNA (mtDNA) and nuclear DNA (nDNA), to serum.

As a result, the cell-free DNA level is studied as a potential biomarker for early diagnosis, diagnosis and prognosis. Regarding cell-free DNA in serum of acute SCI patients, it is crucial to characterize the intrinsic kinetics properties of cell-free DNA, especially when using cell-free DNA analysis as longitudinal diagnostic tool for monitoring the course of treatment. Many studies showed cell-free DNA has short half-time from minutes to hours in different condition, such as uncomplicated singleton pregnancies [29], colorectal cancer [17], exercise-induced inflammation [30–33] and healthy subjects [34, 35].

At present, little is known about the role and time course of circulating cell-free nDNA and changes in mtDNA concentration in patients with acute cervical SCI. We propose that (1) cell-free nDNA and mtDNA is liberated into the plasma early after the onset of acute cervical SCI; (2) associations among the plasma nDNA and mtDNA, disease severity and clinical outcome scores existed in patients with acute cervical SCI. Therefore, our aim was to investigate the relationship between serial circulating cell-free nDNA and mtDNA levels and clinical outcomes in patients with acute cervical SCI.

## Patients and methods

### Patients

Ethical approval for the study protocol was granted by the Research Ethics Committee (103-5218B and 106-1104C). The written informed consent from the participants or their representatives was gathered prior to participation in this study. Patients age 20 to 70 years-old who had suffered from acute blunt traumatic cervical SCI and consecutively admitted to the emergency department (ED) were included in the study. The method of diagnosis for acute traumatic cervical SCI was based on clinical symptoms and spinal imaging, including X-ray, magnetic resonance imaging (MRI) and/or computed tomography (CT). Patients were excluded if (1) they had previous cervical spine trauma or cervical spine surgery; (2) the duration between injury and admission to our hospital was more than 24 h; (3) had severe underlying diseases; or (4) had severe multiple trauma with unstable hemodynamic status.

Thirty patients were excluded from this study, including 11 who refused to participate, two with previous cervical trauma, ten admitted to our hospital more than 24 h after injury, four with severe underlying diseases (three end stage renal failure and one severe liver cirrhosis) and three with severe multiple trauma and unstable hemodynamic status. In total, 44 adult patients who had suffered from acute blunt cervical SCI were included in the study. The mechanisms of injury included 33 traffic accidents, 10 falling accidents, and 1 collision with heavy objects.

For comparison, 66 age- and sex-matched healthy volunteers who received an annual physical checkup were recruited as controls. A clinical interview performed by an experienced neurologist (Lu CH or Tsai WN). These individuals in the control group had similar levels of education but no medical history of brain trauma, cervical injury, neurosurgical intervention of cervical spine, substance abuse, neurological diseases, or psychiatric illnesses. All necessary written informed consents from any healthy volunteers involved in the study was provided, including consents to participate in the study where appropriate.

### Clinical manifestations

The neurological status of patients were recorded at the time of admission using the Japanese Orthopaedic Association (JOA) cervical spine myelopathy functional assessment score [36, 37]. The Abbreviated Injury Score (AIS) for individual body regions was determined and the total extent of the injury was calculated using the objective Injury Severity Score (ISS) upon admission [38]. The choices of operation depended on imaging findings and clinical presentations. The neurological status of each patient was evaluated at admission and at 6 months follow-up according to the JOA disability scale. The neurological recovery rate was calculated using the Hirabayashi method: (6 months follow-up JOA score − at admission score)/(18 − at admission score) × 100% [39]. Recovery rates were graded as follows: ≧ 75%, excellent; 50–74%, good; 25–49%, fair; and < 25%, poor [37]. Outcome was assessed upon 6 months after SCI using the JOA neurological recovery rate, with excellent outcome defined as JOA neurological recovery rate ≧ 75% and non-excellent outcome as JOA neurological recovery rate < 75%.

### Blood sampling and laboratory investigations

Blood samples were taken from 44 SCI patients within 24 h after the onset of injury, at day 14, 30, 90 and 180 post-injury. Sixty-six blood samples from 66 volunteer subjects were collected.

Peripheral venous blood was collected into ethylenediaminetetraacetic acid (EDTA)-containing tubes and was centrifuged immediately at 3000 rpm for 10 min at 4 °C. Then the plasma was removed and placed into a clear tube and frozen at − 20 °C prior to extraction. Blood samples from the control group were collected and processed in the same way. Procedural details were as described previously [40, 41].

According to the manufacturer’s protocol, the DNA was extracted from plasma samples using a QIAamp Blood Kit (Qiagen). Per column, 200 μl of plasma sample was used for DNA extraction. The target DNA concentration in plasma was measured by real-time quantitative polymerase chain reaction (RT-PCR) assay (Roche Lightcycler; Roche, Lewes, UK) for detecting the -globin and ND2 genes as plasma nuclear and mitochondrial DNA [40, 41].

Present study used the primers from human β-globin gene including a β-globin-354F (5′-GTG CAC CTG ACT CCT GAG GAG A-3′) and a β-globin-455R (5′-CCT TGA TAC CAA CCT GCC CAG-3′). The mtDNA were measured by specific primer pair for ND2 (forward: 5′-CAC AGA AGC TGC CAT CAA GTA -3′; reverse: 5′-CCG GAG AGT ATA TTG TTG AAG AG -3′). Both based in several previous studies related with circulating cell-free DNA [12, 14, 19, 20]. Human genomic DNA facilitates the DNA standard curve (Roche, Lewes, UK), a standard curve that includes a positive genomic DNA control and a negative control was repeated. The DNA standard curve and quantitative results are expressed as ng/ml as described previously [14].

### Data analysis

Data in present study were expressed as median (inter-quartile range [IQR]). Categorical variables were analyzed by the Chi-square test or Fisher’s exact test. Continuous variables were analyzed by the Mann–Whitney U test for two groups and were analyzed by the Kruskal–Wallis test followed by the Dunn post-test among three groups. Correlations were analyzed using the Spearman nonparametric correlation method to explore the relationship between age, BMI, laboratory data, JOA score at admission, ISS at admission, and serial plasma DNA (nDNA and mtDNA) levels. All p values presented are two-tailed and the values of p < 0.05 were considered statistically significant.

Assessment of differences in plasma DNA levels in patients with excellent or non-excellent outcomes 6 months after acute cervical SCI using receiver operator characteristics (ROC) plots. The ROC curve was used to estimate an optimal cut-off value for plasma DNA levels for prediction of non-excellent outcome. A cutoff point on the curves was chosen to attain the best compromise between sensitivity and specificity. Areas under the ROC curves (AUCs) were calculated for each parameter and compared. Stepwise logistic regression analysis was performed to eliminate confounding factors and was used to evaluate the relationship between significant variables and clinical outcomes. All of the statistical analyses were conducted using the SAS software package, version 9.1 (2002, SAS Statistical Institute, Cary, North Carolina).

## Results

### Baseline characteristics of the study patients

The baseline characteristics of the 44 adult acute cervical SCI cases and 66 controls were listed in Table 1. The 44 acute cervical SCI patients included 36 males (age range, 19–67 years; median age, 48 years) and 8 females (age range, 23–67 years; mean age, 50 years).

**Table 1:** Demographic data of patients and controls at admission

Parameters	Acute SCI (n = 44)	Controls (n = 66)	*p* value
Age (year), median (IQR)	49.5 (35.5, 58.8)	41.0 (35.8, 46.3)	.084
Male	36	38	.012
Body mass index	24.5 (21.0, 28.2)	24.3 (20.3, 25.5)	.469
Level of SCI			
C1–C4	28	NA	
Below C4	16	NA	
Injury Severity Score at admission			
Total, median (IQR)	17.5 (16, 25.5)	NA	
AIS-head/neck, median (IQR)	4 (4, 4)	NA	
AIS-thorax, median (IQR)	0 (0, 0)	NA	
AIS-abdomen, median (IQR)	0 (0, 0)	NA	
AIS-extremities, median (IQR)	0 (0, 1.75)	NA	
JOA score at admission, median (IQR)	4 (3, 7.5)	NA	
Neurosurgical intervention (n = 39)			
Emergent surgery	23	NA	
Elective surgery	28	NA	
Laboratory data at presentation, median (IQR)			
WBC (× 10^3^/ml)	11.3 (7.67, 15.1)	5.6 (4.92, 7.6)	≤ *0.001*
Platelet counts (× 10^3^/ml)	222 (182, 259)	227 (194, 298)	.238
Plasma DNA at presentation, median (IQR)			
Plasma nuclear DNA (ng/ml)	40.3 (23.2, 96.5)	25.1 (19.45, 38.07)	*.005*
Plasma mitochondrial DNA (ng/ml)	43.6 (20.8, 99.7)	12.6 (8.25, 17.02)	≤ *0.001*

Data are presented either as absolute numbers or as medians with the interquartile range. Statistical significance was set at a level of p = 0.05. Statistical variance between groups was assessed by the Fisher’s exact test for discrete variables and by the Mann–Whitney U test for continuous variables. The italic values reflect p value < 0.05

*SCI* spinal cord injury, *CI* confidence interval, *IQR* interquartile range, *AIS* abbreviated injury scale, *JOA* Japanese Orthopaedic Association, *WBC* white blood cell, *DNA* deoxyribonucleic acid, *NA* not available

Thirty patients were excluded from this study, including 11 who refused to participate, two with previous cervical trauma, ten admitted to our hospital more than 24 h after injury, four with severe underlying diseases (three end stage renal failure and one severe liver cirrhosis) and three with severe multiple trauma and unstable hemodynamic status. The numbers of highest injury level included four at C2, 10 at C3, 14 at C4, seven at C5, six at C6 and three at C7. The majority of injury was between C3 and C4 (54.5%, 24/44). The median (IQR) ISS and JOA score at admission was 17.5 (16, 25.5) and 4 (3, 7.5), respectively. Thirty-nine had cervical spine surgery including 23 emergent surgery within 24 h after SCI and 28 elective surgery. Compared with controls, WBC, plasma nDNA and mtDNA at admission were statistic significantly higher in acute cervical SCI patients (11.3 vs. 5.6, 40.3 vs. 25.1, and 43.6 vs. 12.6; p ≦ 0.001, p = 0.005, and p ≦ 0.001, respectively).

The distribution of injury severity, neuro-surgical intervention and MRI findings at admission of the 44 acute cervical SCI patients were listed in Table 2. There was strongly negative correlated between JOA score and ISS at admission (p ≦ 0.001, r = − 0.591 spearman rho). The most common cervical spinal MRI findings at admission was spinal cord contusion (18/44, 40.9%) and spinal cord edema (11/44, 25%). Of these 23 emergent cervical surgeries, 91.3% (21/23) of patients were severe JOA score, 4.3% (1/23) were moderate, and 4.3% (1/23) were mild. Of these 28 elective cervical surgeries, the majority was ACDF ± corpectomy (89.3%, 25/28). There were no major neurosurgical complications, such carotid/vertebral arteries injury, esophagus perforation, tracheal injury, severe central nerve system infection, or cerebrospinal fluid leakage, except one patient had surgical wound infection in 18 days after SCI. The median (IQR) of JOA score and ISS at admission of those who underwent emergent neurosurgical treatments were 3 (2, 4) and 20 (16, 43), respectively.

**Table 2:** Distribution of Injury severity, neurosurgical interventions and MRI findings at presentation in acute cervical SCI patients

	Severity of traumatic spinal cord injury		
	Severe (n = 33) (JOA score 0–7)	Moderate (n = 5) (JOA score 8–11)	Mild (n = 6) (JOA score 12–15)
Injury level			
C1–C4	23	3	2
Below C4	10	2	4
ISS score at presentation			
0–8	0	0	1
9–15	2	2	2
16–24	20	3	3
> 24	11	0	0
MRI findings at presentation			
Spinal cord swelling	2	3	3
Spinal cord edema	6	2	3
Spinal cord contusion	18	0	0
Intramedullary hemorrhage	7	0	0
Neurosurgical intervention			
Emergent surgery (< 24 h)			
Laminectomy + posterior fixation	18	0	0
ACDF ± corpectomy	3	1	1
Elective surgery (> 24 h)			
Laminectomy + posterior fixation	2	0	0
ACDF ± corpectomy	20	3	2
Laminectomy + ACDF	1	0	0
Plasma DNA at presentation, median (IQR)			
Plasma nuclear DNA (ng/ml)	53.2 (31.5, 104.8)	15.2 (10.0, 82.7)	24.4 (14.9, 26.9)
Plasma mitochondrial DNA (ng/ml)	45.0 (22.3, 137.2)	23.3 (11.4, 135.9)	36.8 (20.8, 99.7)

*JOA* Japanese Orthopaedic Association, *ISS* Injury Severity Score, *MRI* magnetic resonance imaging, *ACDF* anterior cervical discectomy and fusion, *IQR* interquartile range, *DNA* deoxyribonucleic acid

**Fig. 1:**
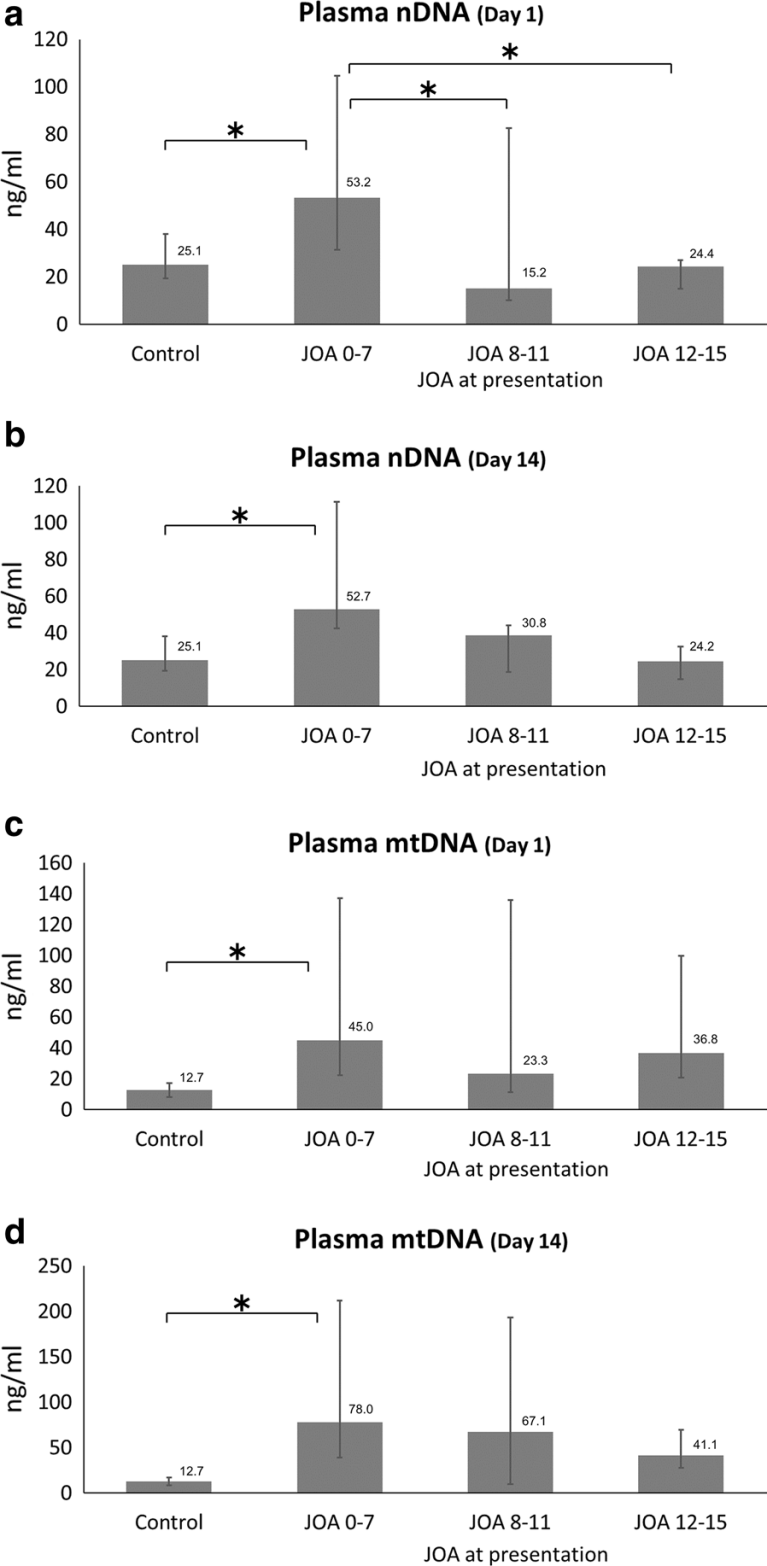
**a**, **b** Plasma nuclear DNA and **c**, **d** plasma mitochondrial DNA levels (present with median and IQR) on Days 1 and 14 in control and in patients with acute cervical SCI stratified by the JOA score on presentation. *Significantly different from control and SCI groups (p ≤ 0.001, Kruskal–Wallis, followed by Dunn test)

Patients with severe JOA score had significantly elevated plasma nDNA levels at admission when compared patients with moderate and mild JOA score (53.2 vs. 15.2 vs. 24.4 ng/ml, *p *= 0.007), but no significant difference in plasma mtDNA levels (45.0 vs. 23.3 vs. 36.8 ng/ml, *p *= 0.647). Median plasma nDNA levels were significantly higher in the severe SCI group (JOA 0–7) at day 1 and day 14 when compared with the control group (53.2 and 52.7 vs. 25.1 ng/ml, p < 0.001) (Fig. 1a, b). Similarly, median plasma mtDNA levels were significantly higher in the severe SCI group (JOA 0–7) at day 1 and day 14 when compared with the control group (45.0 and 78.0 vs. 12.7 ng/ml, p < 0.001) (Fig. 1c, d).

### Association of plasma DNA levels with age, spine injury level, JOA score, ISS, WBC, platelet count, renal function, liver function, and ICU stay in acute cervical SCI patients

Correlation analysis was used to test the correlation plasma nDNA and mtDNA levels at admission on age, spine injury level, JOA score, ISS, WBC, platelet count, renal function, liver function, and length of intensive care unit (ICU) stay. Plasma nDNA level at admission strongly correlated with JOA score, aspartate transaminase (AST), and length of ICU stay (r = − 0.447, *p *= 0.003, r = 0.414, *p *= 0.019, and r = 0.336, *p *= 0.028, respectively). Plasma mtDNA level at admission only correlated with AST level (r = 0.410, *p *= 0.020). However, both plasma nDNA and mtDNA at admission were not correlated with ISS at admission (r = 0.184, *p *= 0.237, and r = 0.160, *p *= 0.306, respectively).

**Fig. 2:**
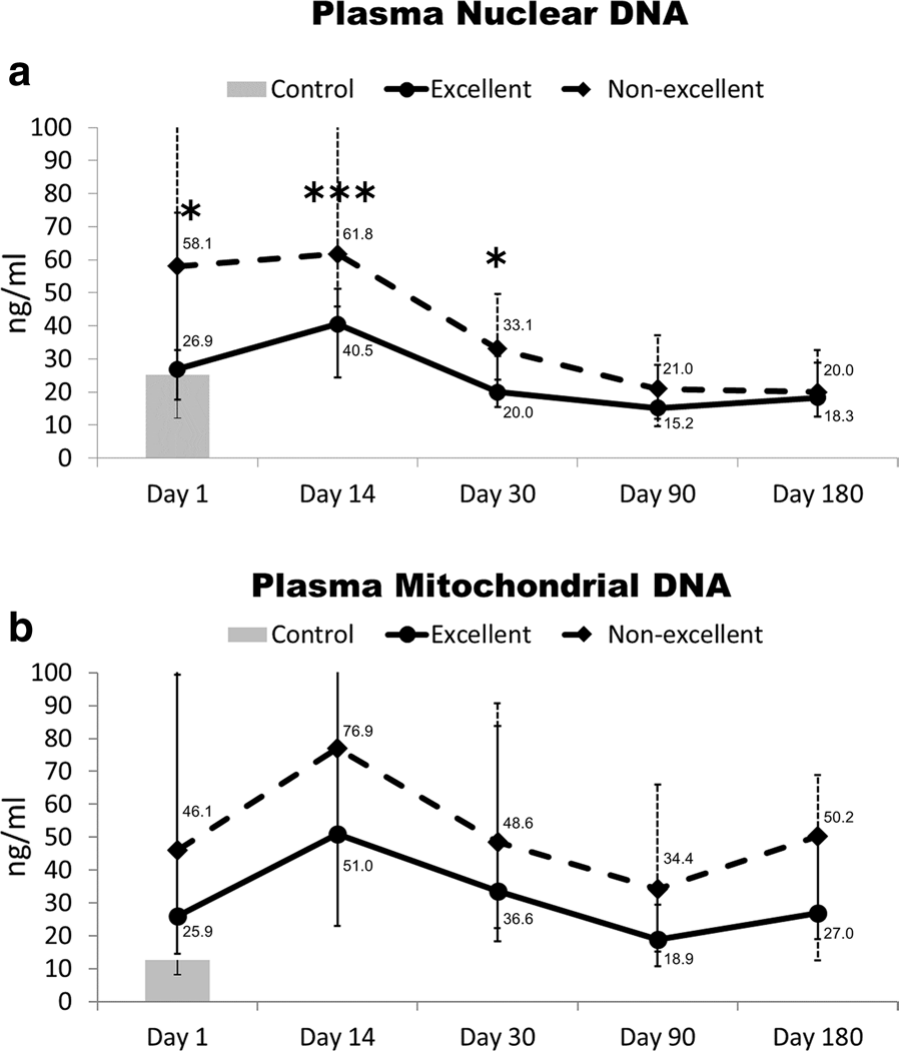
**a** Plasma nuclear DNA and **b** plasma mitochondrial DNA levels (present with median and IQR) on days 1, 14, 30, 90 and 180 in patients with acute cervical SCI and in the controls. *p < 0.05; ***p ≤ 0.001, acute cervical SCI patients with non-excellent outcome vs. those with excellent outcome, by Mann–Whitney U test

By JOA score on day 180 of the 44 acute cervical SCI patients, the median (IQR) recovery rate was 66.7 (26.7, 85.7), including 21 were excellent (≧ 75%), 9 were good (50–74%), 3 were fair (25–49%), and 11 were poor (< 25%). The time course of plasma nDNA and mtDNA concentration changes in acute cervical SCI patients with excellent (JOA recovery rate ≧ 75%) and non-excellent (JOA recovery rate < 75%) outcomes was compared. From day 1 to day 30, plasma nDNA levels were significantly higher in the non-excellent outcome group (median, 58.1 ng/ml, 61.8 ng/ml, and 33.1 ng/ml, respectively) than in the excellent outcome group (median, 26.9 ng/ml, 40.5 ng/ml, and 20.0 ng/ml, respectively) (*p *= 0.018, *p *= 0.001, and *p *= 0.014, respectively) (Table 3). There were no significant difference on day 90 and day 180 (Table 3, Fig. 2a).

**Table 3:** Characteristics of patients with acute cervical spinal cord injury stratified by outcome (excellent/non-excellent)

	Excellent outcome (N = 21)	Non-excellent outcome (N = 23)	p value	Odds ratio	95% CI (lower, upper)
Age (year), median (IQR)	45 (36.5, 56)	53 (31, 63)	.102		
Male	17	19	1.000	1.059	0.488, 2.297
Body mass index	25.2 (21.1, 28.3)	24.3 (20.9, 26.9)	.860		
Level of SCI			1.000	.929	0.494, 1.746
C1–C4	13	15			
Below C4	8	8			
Injury Severity Score at admission					
Total, median (IQR)	16 (13, 21)	21 (16, 43)	.022		
AIS-head/neck, median (IQR)	4 (3, 4)	4 (4, 5)	.040		
AIS-thorax, median (IQR)	0 (0, 0)	0 (0, 1)	.055		
AIS-abdomen, median (IQR)	0 (0, 0)	0 (0, 0)	.591		
AIS-extremities, median (IQR)	0 (0, 2)	0 (0, 1)	.608		
JOA score at admission, median (IQR)	6 (4.5, 12)	4 (2, 4)	≤ .001		
Neurosurgical intervention					
Emergent surgery	6	17	.006	2.738	1.307, 5.734
Elective surgery	12	16	.533	1.313	0.715, 2.411
Laboratory data at presentation, Median (IQR)					
WBC (× 10^3^/ml)	12.9 (9.3, 16.0)	10.0 (6.3, 14.3)	.105		
Platelet counts (× 10^3^/ml)	225 (181, 262)	211 (179, 260)	.724		
Creatinine	0.91 (0.73, 1.0)	0.94 (0.66, 1.05)	.787		
AST	27 (24, 36)	31 (28, 38.3)	.202		
ALT	26 (18, 32)	27 (15, 37)	.814		
Sugar	133 (108, 153)	121 (97, 135)	.231		
Plasma nuclear DNA (ng/ml) at presentation, Median (IQR)					
Day 1	26.9 (17.8, 74.2)	58.1 (32.6, 160)	.018		
Day 14	40.5 (24.4, 51.1)	61.8 (45.9, 139)	.001		
Day 30	20.0 (15.4, 30.9)	33.1 (23.8, 49.7)	.014		
Day 90	15.2 (11.9, 28.2)	21.0 (9.6, 37.1)	.586		
Day 180	18.3 (12.5, 28.2)	20.0 (17.2, 32.6)	.344		
Plasma mitochondrial DNA (ng/ml) at presentation, Median (IQR)					
Day 1	25.9 (14.6, 99.3)	46.1 (26.9, 202.5)	.264		
Day 14	51.0 (23.0, 133)	76.9 (50.7, 168)	.297		
Day 30	33.6 (18.2, 83.9)	48.6 (22.4, 90.7)	.425		
Day 90	18.9 (10.7, 29.6)	34.4 (15.1, 65.9)	.220		
Day 180	27.0 (18.9, 51.8)	50.2 (12.5, 68.8)	.501		
Days of ICU stay	3 (2, 6)	5.5 (3, 16.8)	.026		
Days of neurosurgical stay	12 (4.5, 16.5)	17 (8, 30)	.036		

Data are presented either as absolute numbers or as medians with the interquartile range. Statistical significance was set at a level of p = 0.05. Statistical variance between groups was assessed by the Fisher’s exact test for discrete variables and by the Mann–Whitney U test for continuous variables

*CI* confidence interval, *IQR* interquartile range, *AIS* Abbreviated Injury Scale, *JOA* Japanese Orthopaedic Association, *WBC* white blood cell, *AST* aspartate aminotransferase, *ALT* alanine aminotransferase, *DNA* deoxyribonucleic acid

Plasma mtDNA levels were higher in the non-excellent outcome group (median, 46.1 ng/ml, 76.9 ng/ml, 48.6 ng/ml, 34.4 ng/ml, and 50.2 ng/ml, respectively) than in the excellent outcome group (median, 25.9 ng/ml, 51.0 ng/ml, 33.6 ng/ml, 18.9 ng/ml, and 27.0 ng/ml, respectively). However, there was no statistically significant difference (*p *= 0.264, 0.297, 0.425, 0.220 and 0.501, day 1 to day 180 respectively) (Fig. 2b).

### Outcome and prognostic factors of acute cervical SCI patients

**Fig. 3:**
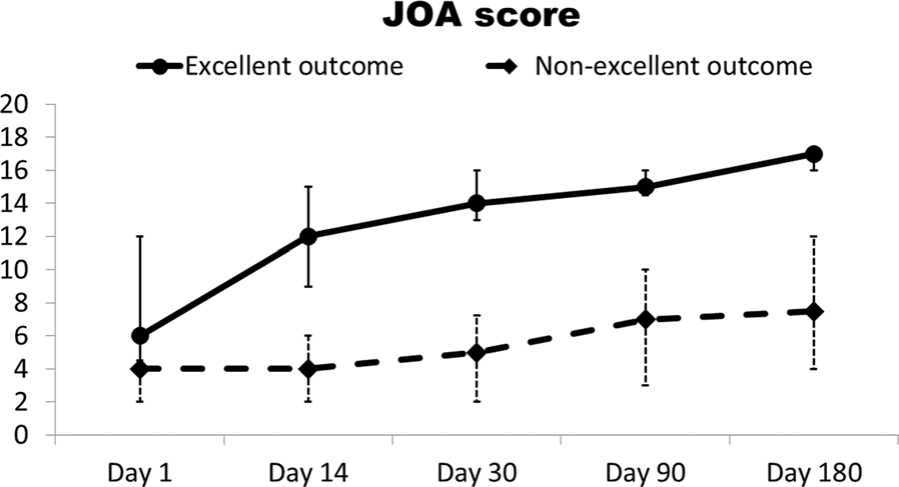
JOA score (present with median and IQR) on days 1, 14, 30, 90 and 180 in patients with acute cervical SCI. ***p ≤ 0.001, acute cervical SCI patients with non-excellent outcome vs. those with excellent outcome, by Mann–Whitney U test

The clinical features and laboratory data of the patient groups with excellent (JOA recovery rate ≥ 75%) and non-excellent (JOA recovery rate < 75%) outcomes were compared (Fig. 3). Statistical analysis revealed significant differences in ISS at admission (*p *= 0.022), AIS-head/neck (*p *= 0.040), JOA score at admission (*p *≤ 0.001), emergent neuro-surgical intervention (*p *= 0.006), plasma nDNA levels at admission, day 14 and day 30 (*p *= 0.018, *p *= 0.001 and *p *= 0.014, respectively), and the length of ICU and hospital stay (*p *= 0.026 and *p *= 0.036, respectively) (Table 3).

All of these variables except for emergent neuro-surgical intervention, the length of ICU and hospital stay were used in the logistic regression analysis. Only JOA score at admission (*p *= 0.011; expectancy: 0.502; 95% CI 0.295–0.853), and plasma nDNA levels on day 14 (*p *= 0.020; expectancy: 1.304; 95% CI 1.005–1.064) were independently associated with clinical outcome.

To determine the relationship between plasma nDNA level on day 14 and outcome, the ROC curves were generated. The AUC for plasma nDNA level on day 14 was 0.817 (*p *= 0.001, 95% CI 0.685–0.950). The cut-off value of plasma nDNA level at admission was 45.6 ng/ml (sensitivity 78.9% and specificity 78.4%).

## Discussion

The time course of plasma DNA levels in patients with acute cervical SCI produced was examined and yielded the following major findings. First, both plasma nDNA and mtDNA levels at admission were significantly higher in SCI patients compared to controls. Patients with severe JOA score at admission had significantly elevated plasma nDNA levels when compared patients with moderate and mild JOA score (53.2 vs. 15.2 vs. 24.4 ng/ml, *p *= 0.007), but no significant difference in plasma mtDNA levels (45.0 vs. 23.3 vs. 36.8 ng/ml, *p *= 0.647). Second, plasma nDNA levels at admission in acute cervical SCI patients were significantly correlated with the JOA score (r = − 0.447, *p *= 0.003), AST level (r = 0.414, *p *= 0.019), and length of intensive care unit stay (r = 0.336, *p *= 0.028). Third, plasma mtDNA levels at admission were only significantly correlated with AST levels (r = 0.410, *p *= 0.020). Fourth, plasma nDNA increased significantly from day 1 to 30 in patients with a poor outcome (Fig. 2a). Moreover, the JOA score at admission and plasma nDNA level on day 14 were independently associated with outcome. Lastly, a cut-off value of 45.6 ng/ml of plasma nDNA level on day 14 is associated with poor outcome in acute cervical SCI patients.

### Increases in plasma DNA in SCI patients are associated with severity of trauma and predict outcomes

Both our previous study [12] and that of Filho et al. [13] reported that the plasma cell-free DNA level at admission was an independent predictor of poor outcome and mortality in patients with acute traumatic brain injury (TBI). In our previous study [12], higher plasma nDNA levels (> 72.95 ng/ml) were associated with poorer outcomes. Filho et al. [13] reported that a higher plasma DNA level (> 171,381 kilogenome-equivalents/l; equal to 1131.1 ng/ml) predicted mortality with 90% specificity and 43% sensitivity in severe TBI patients.

Similar findings have been reported by other studies; [8–13] all individuals who have suffered a severe trauma have elevated plasma concentrations of cell-free DNA at admission compared to healthy individuals. These elevated DNA concentrations correlate with trauma severity and may be useful for predicting outcome [8–13]. It is possible that acute trauma results in tissue damage and the release of DNA into the circulation via necrosis [42, 43] and apoptosis [44, 45]. Macher et al. [10] revealed that plasma DNA levels at 24 h after admission were positively correlated with the ISS and Acute Physiology and Chronic Health Enquiry (APACHE II) score in patients with TBI, but there was no association with the presence of extracranial injury. Yurgel et al. [9] found no significant difference in plasma DNA concentration between patients with isolated TBI versus those with TBI and extracranial injuries.

In the current study, plasma nDNA levels at admission in acute cervical SCI patients were significantly correlated with the JOA score at admission (r = − 0.447, *p *= 0.003). The extent of injury on admission, as evaluated by the ISS, predicted the clinical outcome (*p* = 0.022, Table 3). However, plasma nDNA and mtDNA levels at admission were not correlated with the ISS at admission (r = 0.184, *p *= 0.237, and r = 0.160, *p *= 0.306, respectively). Because we had excluded patients having severe multiple trauma with unstable hemodynamic status and there was no patient had complete spinal cord injury in our study, the injury severity is relative mild in this study (median ISS = 17.5, Table 1). The ISS majorly came from Head/Neck region (spinal cord injury). Although the ISS was higher in poor outcome group (Table 3), the ISS was not significant correlated with nuclear and mitochondrial DNA plasma levels. In Lo et al. study, significant correlations were observed between plasma DNA concentrations and the AIS values for the head and neck region (Spearman rank-order correlation, p < 0.0001; r = 0.440), the thorax (Spearman rank-order correlation, p < 0.001; r = 0.520), and the abdomen (Spearman rank-order correlation, p = 0.0002; r = 0.418). No significant correlation was observed between plasma DNA concentrations and the AIS values for the extremities (Spearman rank-order correlation, p = 0.136; r = 0.165) [11]. The elevated plasma cell-free DNA level seen in SCI patients may be due to its direct release from damaged extraspinal cord tissues and damaged spinal cord tissue through a disrupted brain-blood barrier. Plasma nDNA levels at admission, and on days 14 and 30 (*p* = 0.018, *p* = 0.001 and *p* = 0.014, respectively), were more powerful predictors of outcome than the ISS score at admission (*p* = 0.022). Therefore, in the current study, nuclear and mitochondrial DNA from extraspinal cord injury did not confuse our main results.

### Plasma DNA level is predictive of complications and mortality for different diseases

Previous studies have shown that the plasma DNA level is significantly higher in patients with a severe injury who eventually develop severe complications [8], and is also correlated with mortality [9, 10, 13]. However, both plasma nDNA and mtDNA levels were much lower in our SCI patients (40.3 and 43.6 ng/ml, respectively) compared to cohorts in other studies of severe trauma (560–2418 ng/ml) [8–10, 13], and sepsis patients (436 and 149 ng/ml, respectively [median values]) [15]. Moreover, plasma nDNA and mtDNA levels were similar at admission in our SCI patients (40.3 and 43.6 ng/ml, respectively) to those reported for other brain diseases, such as ischemic stroke [21] (44 and 25 ng/ml), spontaneous intracranial hemorrhage [19] (20 and 10 ng/ml), aneurysmal subarachnoid hemorrhage [20] (56 and 17 ng/ml), bacterial meningitis (100 and 22 ng/ml), and aseptic meningitis [14] (34 and 17 ng/ml).

Previous studies have shown that plasma DNA decreases rapidly in severe TBI patients who survive and have no major complications [8, 9]. Plasma DNA concentrations increase early after injury, but then decrease towards reference values within 3 h in patients with less severe injuries [8]. Yurgel et al. observed a similar pattern, where the plasma DNA concentration (mean concentration 366,485 kilogenomes-equivalents/l; equal to 2419 ng/ml) at study entry fell by 65% after 24 h (mean concentration, 131,708 kilogenomes-equivalents/l; equal to 869 ng/ml) [9]. In the early stage after a primary injury, plasma DNA concentrations vary markedly among patients, probably due to multiple sources of DNA release. In our previous TBI study [12], the poor outcome group had a more than a two-fold higher plasma nDNA level than the good outcome group, as early as day 1.

Another interesting finding of the present study was that plasma nDNA and mtDNA levels increased continuously until day 14 after injury, and then dropped quickly thereafter up to day 30 (Fig. 2). The mechanism underlying the appearance of cell-free DNA in the cerebrospinal fluid (CSF) and circulation is unknown. It is possible that the inflammatory response induces further cell damage and causes gradual DNA release into the CSF and circulation. Inflammation can affect the release of DNA from cells undergoing apoptosis or necrosis, although the nature of this effect may vary depending on the inflammatory stimulus and local cellular events [46]. Previous studies have shown that apoptosis is associated with specific pathological conditions in the CNS, including SCI [44, 45]. Our previous study also showed that the nDNA and mtDNA levels in the CSF, and of nDNA in the circulation, are significantly higher from day 1–14 in patients with spontaneous aneurysmal subarachnoid hemorrhage compared to controls [20]. Furthermore, nDNA levels in the CSF and circulation both increased, and reached a peak on day 4. In the brain lateral fluid percussion brain injury rat model of Perez-Polo, inflammatory events and brain–blood barrier dysfunction were evident as early as 3–6 h after injury, and persisted for 18 days post-injury [47]. Their study implied that injury to the central nervous system can release DNA into the circulation, via a disrupted brain–blood barrier, for up to 18 days post-injury; this is consistent with the present findings.

Another possible reason for DNA release may be that patients undergo treatments, such as cervical spine laminectomy, cervical discectomy and interbody fusion, which can also damage the spinal cord and other tissues. In the present study, 39 patients underwent cervical spine surgery, including 23 emergent cases within 24 h after SCI and 28 elective surgical cases. Of the 28 elective surgical cases, the median (range) number of days elapsed before neurosurgical intervention after SCI was 5 (2–31). The differences in nDNA and mtDNA levels between days 1 and 14 were not significant in patients who did and did not undergo elective surgery (*p* = 0.545 and *p* = 0.889, respectively). Neurosurgical interventions may damage tissues and release DNA into the circulation; however, this was not observed in the present study.

The clearance mechanism of DNA from the circulation is not well understood, although previous studies cited the liver and kidneys as prime candidate organs involved in DNA removal [48]. In the present study, AST levels were significantly correlated with plasma nDNA and mtDNA levels at admission (r = 0.414, *p *= 0.019 and r = 0.410, *p *= 0.020, respectively). However, the AST level was not an independent predictor of outcome in logistic regression analysis. Changes in plasma nDNA may be partly attributable to impaired liver function, but this did not confound the major results of this study.

In the present study, the plasma nDNA level on day 14 was independently associated with outcome, suggesting that the therapeutic window is within the first 14 days after injury. Medalha et al. [49] demonstrated that acute DNA damage extends beyond the spinal cord in SCI rats to affect the blood, liver, and kidneys. Bao et al. [50] administrated a monoclonal antibody against CD11d integrin, an integral leukocyte adhesion protein that reduces oxidative stress-related DNA oxidation after severe compression injury. Their findings provide strong evidence that interventions preventing DNA damage may provide a neurological benefit following SCI.

The current study had several limitations. First, the relatively small sample size and the significant number of analyses performed may have biased the statistical analysis, particularly in terms of inferring causality. Second, although the study showed that higher plasma nDNA levels at admission, and on days 14 and 30, were associated with poor outcome in acute cervical SCI patients, some SCI treatments may damage the spinal cord or other tissues and cause the release of DNA, which may have served as a confounding factor; it is reasonable to postulate that surgical intervention can affect serum DNA levels. Furthermore, when the neurosurgical intervention was included in the multiple logistic regression analysis, it was not independently associated with outcome. Thus, neurosurgical intervention may affect plasma DNA levels but was not a confounder with respect to the major findings of this study, and the maximum likelihood estimates of the coefficients were valid. The sample size for each JOA category was very different [severe (n = 33); moderate (n = 5); mild (n = 6)], which could explain why that nDNA were significantly higher in severe patients when compared to moderate and mild patients. Nonetheless, large-scale prospective studies are warranted to verify the prognostic value of plasma DNA with respect to clinical outcomes.

## Conclusions

In the present study, JOA score at admission and plasma nDNA levels on day 14 were independently associated with clinical outcome. In theory, JOA score performance and plasma nDNA levels reflect the severity of spinal cord injury. Importantly, we demonstrate that plasma nDNA assays can be considered as potential neuropathological markers in patients with acute traumatic cervical SCI and that there may be a therapeutic window within the first 14 days post-injury.

## Data Availability

The datasets used and/or analyzed during the current study are available from the corresponding author on reasonable request.

## Abbreviations

SCI: spinal cord injury
DNA: deoxyribonucleic acid
mtDNA: mitochondrial DNA
nDNA: nuclear DNA
CT: computed tomography
MRI: magnetic resonance imaging
JOA: Japanese Orthopaedic Association
AIS: Abbreviated Injury Score
ISS: Injury Severity Score
RT-PCR: polymerase chain reaction
IQR: inter-quartile range
ROC: receiver operator characteristics
AUCs: areas under the ROC curves
ACDF: anterior cervical discectomy and fusion
ICU: intensive care unit
AST: aspartate transaminase
TBI: traumatic brain injury
APACHE II: Acute Physiology and Chronic Health Enquiry
CSF: cerebrospinal fluid
